# The Quality of Dermatology Match Information on Social Media Platforms: Cross-Sectional Analysis

**DOI:** 10.2196/65217

**Published:** 2025-05-28

**Authors:** Anjali D'Amiano, Jack Kollings, Joel Sunshine

**Affiliations:** 1 Department of Dermatology, Johns Hopkins, 600 North Wolfe Street, Baltimore, MD, 21231, United States, 1 (410) 955-5933

**Keywords:** residency, dermatology match, Reddit, Student Doctor Network, dermatology, information, cross-section, cross-sectional analysis, online, qualifications, misinformation, media, online data, TikTok, online platform, health platform, web platform, online health, health information, social media, digital health, social media posts, online content, health content, social media content, residency program, medical residency, MD, medical school

## Introduction

The transition of United States Medical Licensing Examination (USMLE) Step 1 to pass/fail and a new system for signaling interest in programs have complicated the competitive dermatology match process [1]. Candidates frequently use social media for guidance, but advice on these platforms can be misleading and potentially discourage applicants. Our study evaluates the accuracy of dermatology match information on popular social media sites where medical students, residents, and attendings discuss medicine and residency application processes—Reddit, Student Doctor Network (SDN), and TikTok.

## Methods

### Study Design

In March 2024, we used the search terms “how to match into dermatology” and “advice for the dermatology match process,” identifying 34 sources and corresponding response comments from TikTok (n=10), Reddit (n=14), and SDN (n=10). These sources provided insights into application components, including USMLE scores, research experiences, and rotations, which we compared to official 2022 National Residency Matching Program (NRMP) data (n=348), using 2-tailed Student *t* tests to identify differences in quantitative measures. Representative quotes were qualitatively compared to NRMP data and the Association of Professors of Dermatology (APD), Residency Program Directors Section, Information Regarding the 2023‐2024 Application Cycle guidelines [2]. Inclusion criteria required at least one numeric data point for comparison.

### Ethical Considerations

This study was institutional review board approved (IRB00441663) in alignment with ethical considerations.

## Results

Our analysis revealed that mean Step 1 scores (mean 248.0, SD 7.0 vs mean 254.5, SD 8.28; *P*<.001); the number of abstracts, posters, and publications (mean 20.9, SD 3.0 vs mean 23.3, SD 8.68; *P*=.004); and total publications (mean 7.0, SD 1.0 vs mean 13.2, SD 5.65; *P*<.001) reported on the web were significantly higher than NRMP data (Table 1). The NRMP and web-based data did not significantly differ in mean Step 2 scores (mean 257.0, SD 8.5 vs mean 261.0, SD 10.1; *P*=.06).

**Table 1.** Significant differences in self-reported web-based data and National Residency Matching Program (NRMP) data.

Category	NRMP data, mean (SD)	Web-based data, mean (SD)	*P* value^a^
Step 1 score	248.0 (7.0)	254.5 (8.28)	<.001
Step 2 score	257.0 (8.5)	261.0 (10.1)	.06
Number of abstracts, posters, and publications	20.9 (3.0)	23.3 (8.68)	.004
Total publications	7.0 (1.0)	13.2 (5.65)	<.001

a *P* values were calculated by using Student *t* tests.

Representative quotes are found in Table 2. Regarding academic performance, 15 sources addressed medical school grades, with 10 (67%) emphasizing the importance of Alpha Omega Alpha (AOA) status; however, per NRMP data, only 39.7% of matched dermatology residents were members of AOA. Of 21 sources, 19 (90%) recommended participation in away rotations; 11 (52%) provided a specific number, averaging 3.9 rotations, while 8 (38%) suggested completing as many as possible—a contradiction to APD guidelines, which recommend a maximum of 2 external rotations for students with home dermatology programs and 3 for those without such programs [2].

**Table 2.** Representative quotes from categories of dermatology match discussion.

Category	Representative quotes
Research year	“Definitely take a research year to maximize chances, of people I know who didn’t match, most had not taken years.” “A research year is not necessary, there are applicants that we have ranked very highly who have had 3‐5 listed publications and ones we have ranked near the bottom of the list with > 25 publications.” “Taking a research year will help you stand out, build connections, and be productive.” “This is a personal choice, however, and one that must be made after weighing the risks versus benefits. Some program directors I have spoken to do not feel an extra year of research can add much to an applicant’s curriculum vitae, while others feel it is important.” “You don’t need to take one if you had research experiences during medical school, start early.”
Letters of recommendation	“Find big names in dermatology (chairs, PDs^a^, renound [*sic*] researchers, etc).” “3 dermatologists with big names, 1 from any field.” “All three were high-ranking, high-impact, quality letters. As most people will tell you, the fourth letter should come from an away rotation.” “Most applicants will have 1‐2 letters from a non-derm setting (usually medicine sub-internship, research mentor) and 2‐3 derm letters. Pick your letter writers carefully as some attendings can be great clinically but write lackluster letters. Big names on the letter are helpful, but not if they don’t know you well enough to comment on your performance as a student or personal characteristics.”
Rotation grades	“Honor as many preclinical/clinical grades as possible; AOA^b^ is more important than GHHS^c^ but try for both.” “Honors as many rotations as you can. Do well on your dermatology elective.”
Away rotations	“If you do not have a home dermatology department do as many away rotations as possible.” “Do away rotations at programs where they often extend interview invites to interviewers.” “Away rotations are not needed if coming from a top institution.”
Interests and activities	“Try to show only interest in dermatology.” “Programs love it when you’re super specialized and focused and passionate about one or two things and make that your theme of your app. You look a lot cooler and desirable vs the person who tried to do everything and anything in derm to pad their app. HAVE A FOCUS!!” “Extracurriculars aren’t as big a part of the dermatology application process.”
Interview	“The most important part of the match process.”
Signals	“Do not expect to get interviews at places you do not signal.”
Personal statement	“This is not a big part of the process.” “Personal statements will not make or break your application.”
DO^d^ match	“Probably next to impossible to match if you are a DO.”
Medical school	“Try to go to a top 10 medical school if you’re thinking derm.” “Go to [a medical school] with a strong home dermatology department.”

a PD: program director.

b AOA: Alpha Omega Alpha.

c GHHS: Golden Humanism Honors Society.

d DO: Doctor of Osteopathic Medicine.

Web-based sources were also divided about the utility of a research year. Of the 22 sources discussing research years, 16 (73%) supported taking a research year to increase applicants’ number of research experiences, and 6 (27%) articles advocated against taking a research year to match, without a genuine underlying interest in the research.

## Discussion

This study is the first to systematically evaluate the veracity of dermatology match–related discussions that occurred across multiple social media platforms after the USMLE Step 1 pass/fail change. Previous studies on self-reported SDN and Reddit data showed mixed results; a 2017 study found that radiology applicants who self-reported on SDN were likelier to be AOA members with higher USMLE step scores, indicating a reporting a bias toward stronger applicants, which is likely reflected in our study as well [3]. In contrast, a 2020 study found no significant difference in self-reported dermatology applicant USMLE step 1 and 2 scores between social media and NRMP data [4]. However, these studies predate the USMLE Step 1 pass/fail change, and they did not specifically examine forum discussions directly. Our study expands the scope by including TikTok—a platform that is increasingly being used for medical education among students [5].

Our findings suggest potential biases in self-reported data on social media when compared to official sources, underscoring the need for cautious interpretation. Our limitations include the inherent self-reporting nature of social media, which may not accurately reflect the broader applicant pool. Although many contributors on the web aim to help others, some may exaggerate requirements or overstate match difficulties to discourage competition.

In conclusion, while social media serves as a widely used resource for dermatology applicants, it is often unreliable. Program director surveys could help clarify common misconceptions, and efforts to correct misinformation through trusted sources may improve the accuracy of information available to applicants. Applicants seeking reliable guidance should turn to established mentorship programs, such as the National Mentorship Match through the Dermatology Interest Group Association, and official recommendations from the APD. By providing structured, accurate resources, programs can help counter misinformation and better support future applicants.

## Abbreviations

AOA: Alpha Omega Alpha
APD: Association of Professors of Dermatology
NRMP: National Residency Matching Program
SDN: Student Doctor Network
USMLE: United States Medical Licensing Examination

## References

[1] Parker T, Brown AE, Messer A, Lewis GD (2020). Response to: “Reliability of self-reported data on social media vs National Residency Match Program charting outcomes for dermatology applicants”. J Am Acad Dermatol.

[2] (2023). Association of Professors of Dermatology, Residency Program Directors Section, Information Regarding the 2023-2024 Application Cycle. Association of Professors of Dermatology.

[3] Sura K, Wilson LD, Grills IS (2017). Comparison of self-reported data on Student Doctor Network to objective data of the National Resident Matching Program. J Am Coll Radiol.

[4] Hu S, Laughter MR, Dellavalle RP (2020). Reliability of self-reported data on social media versus National Residency Match Program charting outcomes for dermatology applicants. J Am Acad Dermatol.

[5] Lacey H, Price JM (2023). #MedEd-The “TikTok” frontier of medical education. Clin Teach.

